# Comparative effectiveness of digital versus face-to-face cognitive behavioral therapy for alcohol use disorder: a systematic review and meta-analysis

**DOI:** 10.1017/S0033291725102043

**Published:** 2025-10-20

**Authors:** Ji Eun Kim, Jiyeong Kim, Nayeon Choi, Sang Kyu Lee, Hong Seok Oh, Sungwon Roh

**Affiliations:** 1 Department of Psychiatry, Hanyang University College of Medicine, Seoul, Korea; 2 Department of Psychiatry, Hanyang University Hospital, Seoul, Korea; 3 Department of Pre-Medicine, Hanyang University College of Medicine, Seoul, Korea; 4 Biostatics Lab, Medical Research Collaborating Center, Industry–University Cooperation Foundation, Hanyang University, Seoul, Korea; 5 Department of Psychiatry, Hallym University Chuncheon Sacred Heart Hospital, Chuncheon, Korea; 6 Department of Psychiatry, Konyang University College of Medicine, Daejeon, Korea

**Keywords:** alcohol use disorder, cognitive behavioral therapy, digital interventions, face-to-face interventions

## Abstract

Alcohol use disorder (AUD) is a chronic condition that impairs health and function. Cognitive behavioral therapy (CBT) is an evidence-based treatment traditionally delivered face-to-face. Recently, digital CBT delivered online has gained prominence because of access barriers and user preferences. Although many digital CBT studies have emerged, few systematic reviews have directly compared digital and face-to-face CBT in adults with AUD. This systematic review and meta-analysis aimed to evaluate their comparative effectiveness. Following Preferred Reporting Items for Systematic Reviews and Meta-Analyses (PRISMA) 2020 guidelines, a comprehensive search identified 25 randomized controlled trials (*n* = 2,065) comparing these formats. A random-effects meta-analysis evaluated pre- and post-effectiveness by calculating the standardized mean change using raw score standardization (SMCR). For drinking quantity, digital CBT showed a significant pre–post effect (SMCR = 1.21, 95% confidence interval [CI]: 0.38 to 2.04; *p* = 0.004). Face-to-face CBT showed no overall significant effect (SMCR = 0.69, 95% CI: −0.16 to 1.53; *p* = 0.110). However, subgroup analysis of face-to-face trials showed significance for active treatment (SMCR = 1.09), but a nonsignificant negative effect for relapse prevention (SMCR = −0.72). For drinking frequency, both interventions yielded statistically significant effects; however, face-to-face CBT demonstrated a stronger effect (SMCR = 1.02, 95% CI: 0.30 to 1.74; *p* = 0.006) than digital CBT (SMCR = 0.54, 95% CI: 0.29 to 0.79; *p* < 0.001). Forest plots were generated, and Begg’s test was used to assess publication bias.

## Introduction

Alcohol use disorder (AUD) is a major and growing public health concern worldwide. In 2021, an estimated 111.12 million individuals globally were living with AUD, marking a 14.66% increase since 2000 (Danpanichkul et al., 2024). Harmful alcohol use ranks among the leading causes of premature mortality and contributes to approximately 4.7% of the total global disease burden (World Health Organization [WHO], 2024). Despite this substantial burden, treatment coverage for AUD remains critically low; globally, only about 7% of individuals with AUD (and other substance use disorders) who need treatment receive even minimally adequate care (WHO, 2024). This wide treatment gap underscores the urgency of expanding access to effective AUD interventions on a broader scale.

Cognitive behavioral therapy (CBT) is a well-established, evidence-based treatment modality for AUD. It is among the most extensively evaluated psychosocial interventions for substance use disorders, with robust evidence supporting its effectiveness for alcohol dependence. Major clinical guidelines – including those from the National Institute on Alcohol Abuse and Alcoholism (2021) – recommend CBT as a first-line psychosocial treatment for individuals with AUD. By training patients to identify high-risk situations, modify maladaptive drinking-related thoughts, and develop healthier coping strategies, CBT can significantly reduce alcohol consumption and relapse rates. Given its demonstrated efficacy, face-to-face CBT delivered by trained clinicians has long been a cornerstone of AUD treatment in traditional clinical settings.

In recent years, there has been a rapid expansion of digital CBT approaches for mental health and addiction, including AUD. Specifically, the COVID-19 pandemic catalyzed a surge in telehealth and internet-based treatments, as clinicians and patients turned to remote delivery out of necessity (Appleton, Williams, Vera San Juan, Needle, & Schlief, 2021). Digital CBT platforms offer notable advantages in accessibility and scalability: they can deliver therapy to individuals who might not otherwise receive treatment owing to geographic, time, or stigma barriers and can serve large populations with limited incremental costs (Sundström et al., 2022). For example, therapist-guided internet-delivered CBT (iCBT) programs have been shown to effectively reduce alcohol consumption in adults with AUD (Kiluk et al., 2024). Through telemedicine (video conferencing or text-based communication) and self-directed online modules, digital interventions enable patients to receive evidence-based care at their convenience. These benefits have fueled an increasing demand for digital mental health interventions, positioning iCBT as a promising strategy to help bridge the AUD treatment gap at the population level (Sundström et al., 2023).

With the growing adoption of digital CBT, an important clinical question is how its effectiveness compares to the traditional face-to-face format. If digital CBT can match the clinical outcomes of in-person therapy, it could fundamentally improve treatment reach and efficiency; however, any differences in efficacy or patient response between modalities need to be understood to optimize care. Until recently, data directly comparing digital versus face-to-face CBT for AUD have been limited. However, emerging evidence indicates that iCBT can produce therapeutic outcomes comparable to those of conventional delivery. For instance, a randomized clinical trial found that iCBT was noninferior to face-to-face CBT in reducing symptoms of alcohol misuse, suggesting potential applicability to AUD treatment (Sundström et al., 2022). Notably, that study reported no meaningful differences in overall symptom reduction between formats, although the internet-based intervention fell short on certain secondary measures relative to in-person therapy. Such findings indicate that, while the core efficacy of CBT may be retained online, nuanced differences could exist in how each modality impacts specific dimensions of behavior. Indeed, questions remain regarding whether digital interventions truly replicate all the benefits of face-to-face treatment or if unique strengths and limitations exist for each format. Clarifying these issues is clinically important, especially in light of the surging interest in digital care, as it would inform practitioners and policymakers about the appropriate role of digital CBT in AUD treatment.

In this context, the present study aimed to compare the effectiveness of digital and face-to-face CBT in adults with AUD through a systematic review and meta-analysis. To enhance generalizability, we targeted a broad adult population without restricting the sample to a specific region or subgroup. Rather than assuming equivalent outcomes, we sought to identify how each modality performed across distinct behavioral targets. Particular attention was paid to two critical outcome domains – drinking frequency and quantity – as these represent distinct behavioral goals: reducing how often one drinks versus how much one drinks on drinking occasions. The rationale for separating these domains stems from prior research that indicated differential responsiveness depending on the mode of CBT delivery (Kiluk et al., 2024; Sundström, Gajecki, Johansson, & Berman, 2021). Our findings revealed that digital CBT produced a statistically significant reduction in the quantity of alcohol consumed; however, both modalities demonstrated significant effects in reducing the frequency of drinking, with a greater effect size for face-to-face CBT. These results underscore the importance of evaluating the specific strengths of each delivery format and support the potential utility of blended or tailored approaches to optimize AUD treatment.

## Methods

### Protocol and registration

**Figure 1.**
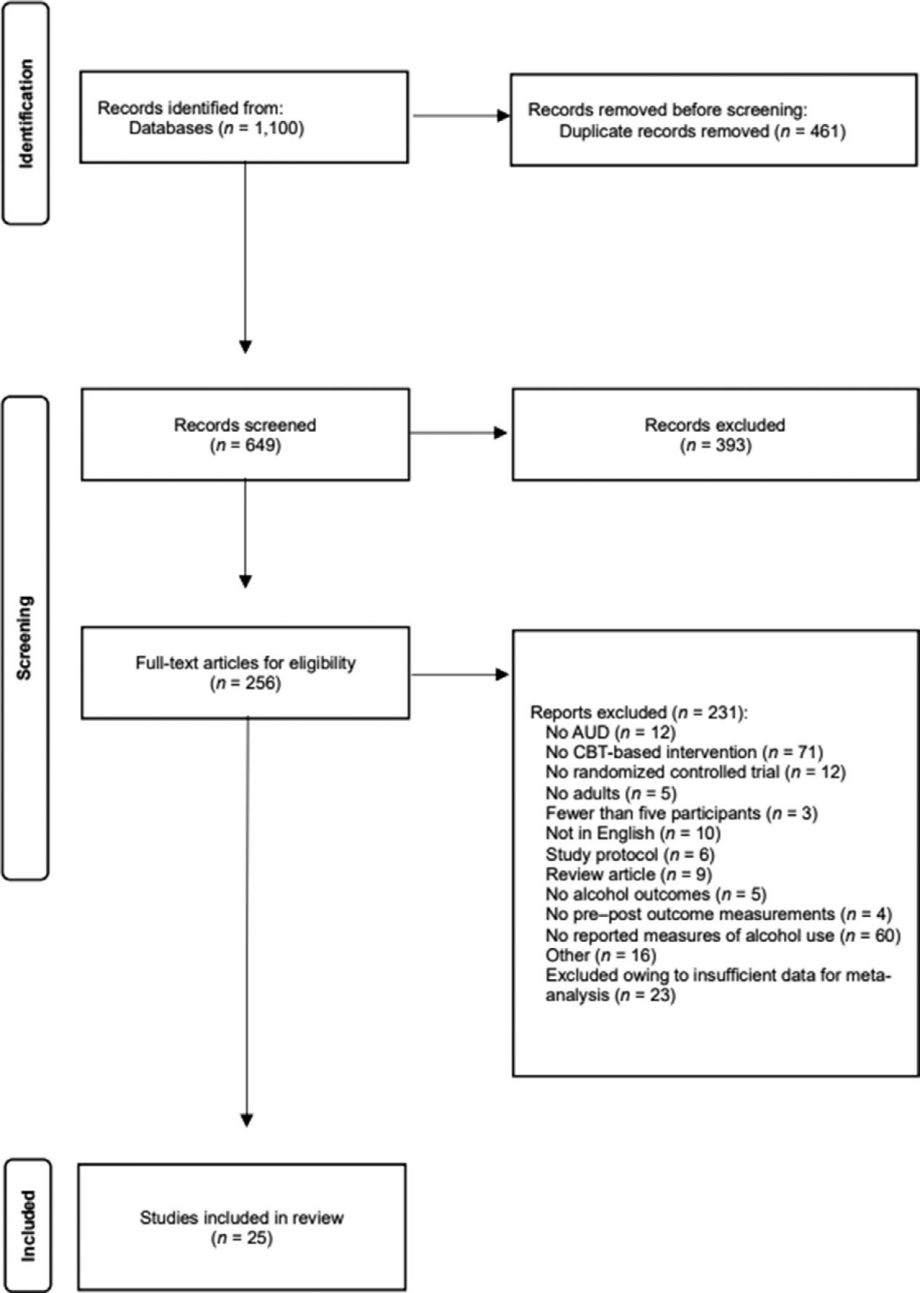
PRISMA 2020 flow diagram illustrating the study selection process. A total of 1,100 records were identified, of which 25 studies met the eligibility criteria and were included in the meta-analysis.

This systematic review and meta-analysis were conducted in accordance with the Preferred Reporting Items for Systematic Reviews and Meta-Analyses (PRISMA) 2020 guidelines (Page et al., 2021). The study selection process is illustrated in Figure 1. The study protocol was prospectively registered in PROSPERO (registration number: CRD42023440071). The methodology adhered to the standard procedures outlined in the Cochrane Handbook for Systematic Reviews of Interventions (Higgins et al., 2022).

### Eligibility criteria

Eligible studies were randomized controlled trials published in peer-reviewed journals, written in English, and available in a full-text format. Studies were included if they examined digital or face-to-face CBT for adults (aged ≥20 years) formally diagnosed with AUD, based on any edition of the Diagnostic and Statistical Manual of Mental Disorders. To be included, studies had to report pre- and post-treatment outcomes related to alcohol use, such as drinking frequency or quantity, to allow for within-group effect size calculation.

### Information sources and search strategy

A systematic search was conducted in PubMed, Embase (via Ovid), and Cochrane CENTRAL. Although the initial protocol included CINAHL and PsycArticles, access to these databases was not available through institutional subscriptions at the time of the search. The search included articles published up to July 2023.

The search terms combined controlled vocabulary (e.g. MeSH terms) and free-text keywords related to “alcohol use disorder,” “cognitive behavioral therapy,” and “digital” or “face-to-face” interventions. Boolean operators (AND, OR) were used to optimize results. The full search strategy is provided in Supplementary Table S2.

### Study selection

Titles and abstracts were screened by a single reviewer (JEK). Full texts of potentially eligible studies were then independently assessed for inclusion by two reviewers (JK and SR). Disagreements were resolved through discussion until a consensus was reached. Study-level characteristics and references are presented in Supplementary Table S1.

### Data extraction and coding

Initial data coding was performed by one reviewer (JEK), followed by collaborative data extraction by three reviewers (JEK, JK, and NC). The extracted information included the study design, sample size, participant demographics, intervention format, outcome measures, and follow-up duration. All data were entered into a standardized Excel spreadsheet. Discrepancies were resolved through group consensus.

### Risk of bias assessment

Risk of bias for each included study was assessed using the revised Cochrane Risk of Bias tool for randomized trials, evaluating five domains: (1) bias from the randomization process, (2) deviations from intended interventions, (3) missing outcome data, (4) outcome measurement, and (5) selection of the reported result. Studies were rated as having a low risk of bias, some concerns, or a high risk, following Cochrane recommendations (Sterne et al., 2019). A visual summary of the risk of bias assessments is provided in Supplementary Figure S1.

### Statistical analysis

The basic characteristics of digital and face-to-face intervention studies were examined. Continuous variables were presented as mean ± standard deviation, and differences between digital and face-to-face intervention studies were assessed using the independent *t*-test or Wilcoxon rank-sum test, depending on the normality of the data distribution. The standardized mean change using raw score standardization (SMCR) was analyzed to assess the effectiveness of digital and face-to-face intervention studies from pre- to post-treatment. For each outcome variable of the digital and face-to-face intervention studies, we plotted forest plots and presented effect sizes with 95% confidence intervals (CIs). Heterogeneity was evaluated using Cochran’s Q test and Higgins’s I^2^ statistic, and a random-effects model was applied according to the results. Publication bias was assessed using Begg’s test. Statistical significance was defined as *p* < 0.05, and all analyses were performed using R version 4.3.0 (R Foundation for Statistical Computing, 2023).

## Results

### Study characteristics

A total of 25 randomized controlled trials were included. Of these, 13 face-to-face and 8 digital CBT studies reported drinking quantity outcomes, and 4 studies per group assessed drinking frequency. For drinking quantity outcomes, the average number of participants per study was significantly higher in the digital CBT group than in the face-to-face group (144.5 ± 223.5 vs. 65.4 ± 62.6, respectively; *p* < 0.001) (Table 1). There was no significant difference in the mean age between the groups (*p* > 0.05), but the proportion of male participants was significantly lower in digital CBT studies (42.2% vs. 65.1%; *p* = 0.011). This corresponded to a female proportion of 57.8% in the digital CBT group and 34.9% in the face-to-face CBT group. Although it was higher in the digital trials, the difference did not reach statistical significance. The intervention completion rate was significantly lower in the digital CBT group (80.8% vs. 92.9%; *p* < 0.001), and the mean follow-up duration was also shorter (8.1 ± 7.1 months vs. 12.2 ± 9.3 months; *p* < 0.001). While the average weekly alcohol consumption tended to be lower in the digital CBT group (312.5 ± 67.6 g vs. 436.2 ± 311.6 g), this difference was not statistically significant (*p* = 0.161). By contrast, among the studies reporting drinking frequency, participants in the digital CBT group had significantly fewer drinking days per week than those in the face-to-face group (3.2 ± 0.3 vs. 5.6 ± 2.0, respectively; *p* = 0.029) (Table 1).

**Table 1.** Characteristics of digital and face-to-face intervention studies

	Drinking quantity			Drinking frequency		
	Face-to-face	Digital	*p*	Face-to-face	Digital	*p*
Number of samples	13	8		4	4	
Number of patients	65.4 ± 62.6	144.5 ± 223.5	< 0.001	81.3 ± 64.3	28.5 ± 21.2	0.200
Age	42.8 ± 3.6	42.3 ± 9.9	0.867	39.8 ± 1.0	37.1 ± 13.5	0.764
Ratio of male patients	65.1 ± 18.6	42.2 ± 9.5	0.011	61.4 ± 2.5	67.4 ± 27.1	0.738
Ratio of patients completing the intervention	92.9 ± 12.7	80.8 ± 18.1	< 0.001	91.9 ± 16.1	90.9 ± 14.3	< 0.001
Follow-up duration (months)	12.2 ± 9.3	8.1 ± 7.1	< 0.001	7.9 ± 4.9	4.0 ± 2.4	0.222
Alcohol consumption per week (g)	436.2 ± 311.6	312.5 ± 67.6	0.161	–	–	–
Drinking days per week	–	–	–	5.6 ± 2.0	3.2 ± 0.3	0.029

### Effectiveness of face-to-face versus digital CBT

The meta-analysis of pre–post intervention effects revealed differing patterns of effectiveness between digital and face-to-face CBT interventions.

**Figure 2.**
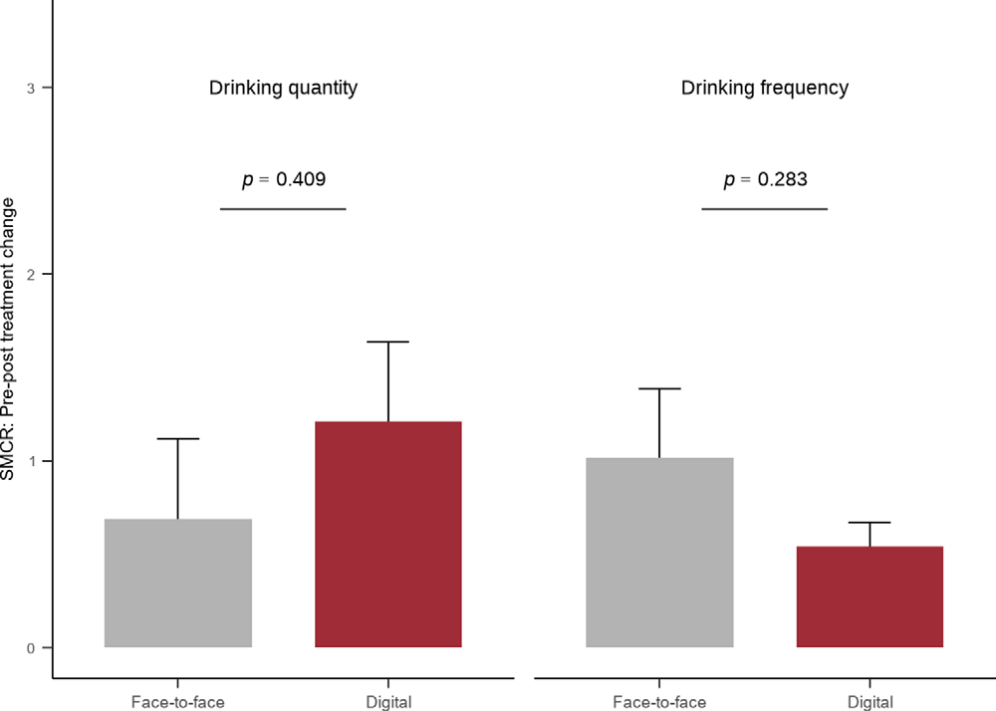
Comparison of the effectiveness of digital and face-to-face interventions on alcohol-related outcomes (bars represent standard errors).

For drinking quantity, face-to-face interventions (*k* = 13) resulted in a nonsignificant SMCR (0.69; 95% CI: −0.16 to 1.53; *p* = 0.110). By contrast, digital interventions (*k* = 8) showed a statistically significant reduction in alcohol consumption (SMCR = 1.21; 95% CI: 0.38–2.04; *p* = 0.004). The difference in effect sizes between intervention methods was not statistically significant (*p* = 0.409). For drinking frequency, face-to-face interventions (*k* = 4) demonstrated a significant effect (SMCR = 1.02; 95% CI: 0.30–1.74; *p* = 0.006), and digital interventions (*k* = 4) produced a statistically significant effect (SMCR = 0.54; 95% CI: 0.29–0.79; *p* < 0.001). However, the difference in SMCR between the two interventions was not statistically significant (*p* = 0.283). These findings suggest modality-specific patterns of within-group effectiveness. Digital CBT showed a significant reduction in the quantity of alcohol consumed, whereas both modalities were effective in reducing the frequency of alcohol use; the effect size for face-to-face CBT was larger, but the between-group differences were not statistically significant (Table 2 and Figure 2).

**Table 2.** Meta-analytic results for pre–post changes by intervention type

	Face-to-face interventions			Digital interventions		
	*K*	SMCR (95% CI)	*p*	*K*	SMCR (95% CI)	*p*
Drinking quantity	13	0.69 (−0.16, 1.53)	0.110	8	1.21 (0.38, 2.04)	0.004
Drinking frequency	4	1.02 (0.30, 1.74)	0.006	4	0.54 (0.29, 0.79)	< 0.001

**Figure 3.**
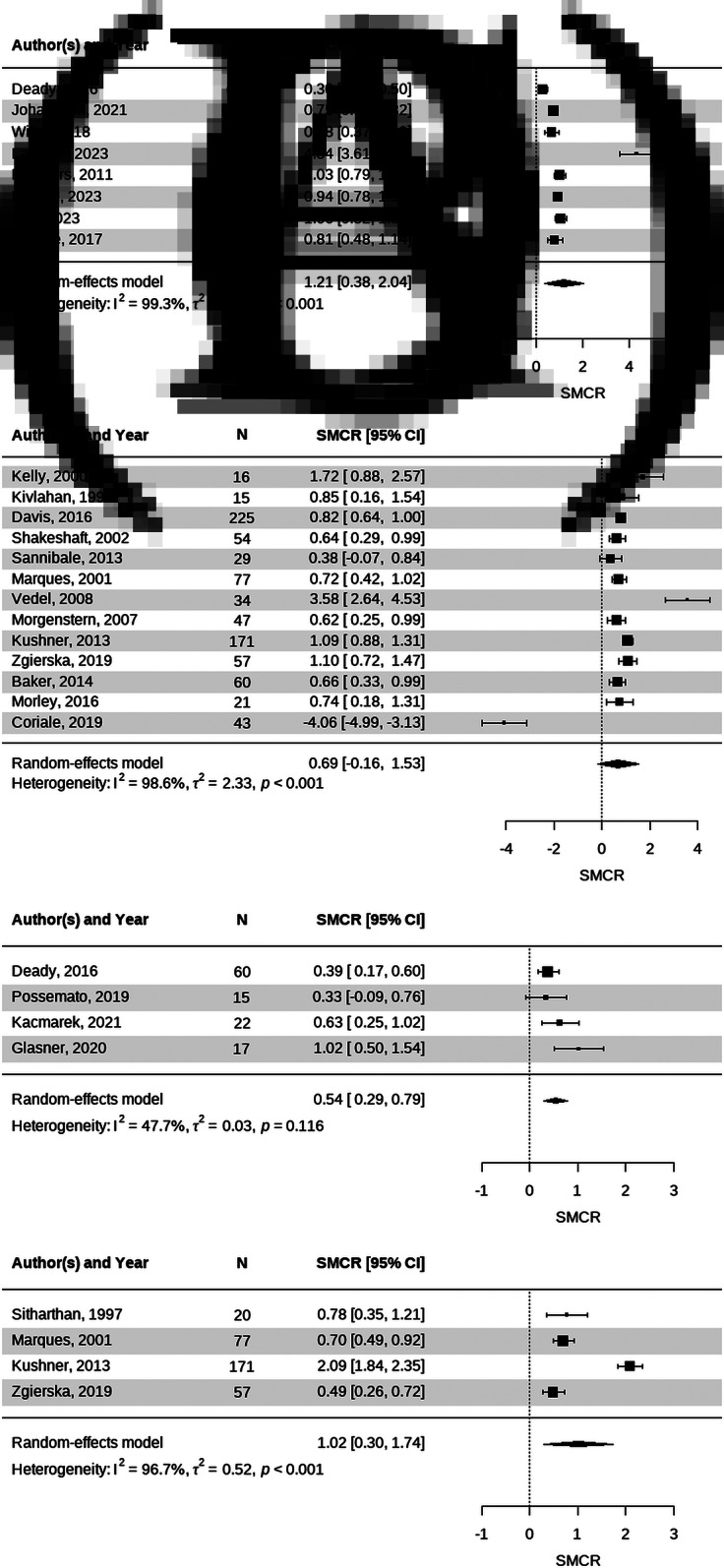
Forest plots of drinking quantity from (A) digital and (B) face-to-face studies and drinking frequency from (C) digital and (D) face-to-face studies.

For drinking quantity, digital interventions showed an overall effect size of 1.21 with high heterogeneity (I^2^ = 99.3%), and face-to-face interventions had an effect of 0.69 with high heterogeneity (I^2^ = 98.6%). Regarding drinking frequency, digital interventions had an effect of 0.54 with moderate heterogeneity (I^2^ = 47.7%), whereas face-to-face interventions showed an effect of 1.02 with high heterogeneity (I^2^ = 96.7%) (Figure 3).

Regarding publication bias, no significant bias was found for either drinking quantity or frequency. In digital interventions, Kendall’s tau was 0.357 (*p* = 0.275) for drinking quantity and 0.667 (*p* = 0.333) for drinking frequency. In face-to-face interventions, Kendall’s tau was −0.026 (*p* = 0.952) for drinking quantity and 0.333 (*p* = 0.750) for drinking frequency.

**Subgroup Analysis by Treatment Goal.** Within face-to-face CBT trials, subgroup analysis of drinking quantity outcomes showed that active treatment studies demonstrated a significant effect (SMCR = 1.09), whereas relapse prevention studies yielded a nonsignificant negative effect (SMCR = −0.72). This suggests that the overall nonsignificant effect of face-to-face CBT on drinking quantity was largely driven by relapse prevention trials with high baseline abstinence. A subgroup analysis of drinking frequency was not feasible because the relapse prevention studies did not report frequency outcomes. The corresponding results are presented in Supplementary Figures S3 and S4.

## Discussion

In this systematic review, we set out to test the hypothesis that digital CBT for alcohol use would not be markedly less effective than face-to-face CBT. The findings largely support this premise. We found that digital CBT was associated with a statistically significant reduction in the amount of alcohol consumed, whereas face-to-face CBT showed a reduction that did not reach statistical significance. By contrast, drinking frequency declined significantly with both interventions, although the effect size was larger for face-to-face CBT (Andersson, Cuijpers, Carlbring, Riper, & Hedman, 2014; Riper et al., 2014a). These results suggest that, while each modality is effective, it may influence different aspects of drinking behavior to varying degrees.

One possible interpretation of the greater impact of digital CBT on consumption volume could be related to the unique features of digital interventions. Online or app-based CBT programs often incorporate structured self-monitoring, personalized feedback, and normative comparisons about alcohol use (White et al., 2010). For example, digital platforms can provide immediate feedback on the number of drinks consumed and how it compares to the recommended guidelines or peer averages, heightening individuals’ awareness of their intake. Such real-time tracking and feedback may empower participants to actively moderate the quantity of alcohol consumed on drinking occasions (Riper et al., 2011). In addition, digital CBT modules can be accessed on demand, allowing individuals to revisit coping strategies or motivational exercises at the exact moments of temptation (e.g. before an evening out), thereby helping curb excessive drinking in the moment. These factors could explain why participants receiving digital CBT achieved larger reductions in total alcohol intake per week.

Conversely, the finding that face-to-face CBT yielded a larger reduction in drinking frequency may indicate advantages inherent to traditional therapy settings. In-person CBT involves direct human interaction, accountability to a therapist, and the therapeutic alliance – elements that can strongly motivate behavior changes (Andersson et al., 2014). Patients who engage in regular face-to-face sessions may feel more accountable to report progress at the next meeting, which can encourage them to stick to planned nondrinking days or avoid high-risk situations, thus reducing how often they drink. Moreover, therapists can dynamically tailor sessions to address emerging issues (e.g. upcoming social events or stressors) and reinforce commitment to abstinence or moderation goals. The rapport and support from a live therapist may instill greater confidence and commitment in patients to skip drinking occasions. Prior research indicates that face-to-face interventions often foster a stronger working alliance and higher treatment engagement than digitally delivered therapy (Sundström, Blankers, & Khadjesari, 2017). Such engagement could impact drinking frequency, for instance, by helping individuals maintain motivation to remain abstinent on more days of the week. These considerations make it plausible that the interpersonal context of face-to-face CBT drives a stronger effect on reducing the frequency of alcohol use, even if the total volume per week is similarly reduced by digital means.

Our results align with a growing body of literature suggesting that technology-delivered CBT can achieve outcomes comparable to traditional in-person therapy for substance use disorders (Riper et al., 2014b; Tait, Spijkerman, & Riper, 2013). Several meta-analyses have established that internet-based interventions produce significantly greater reductions in alcohol consumption compared with no-treatment controls. Notably, a recent large randomized controlled trial demonstrated that therapist-guided iCBT was noninferior to face-to-face CBT in reducing weekly alcohol intake among adults with AUD (Andersson et al., 2014). This finding was further reinforced by Kiluk et al. (2024), who reported that a digital CBT program produced clinically comparable reductions in alcohol use relative to standard face-to-face therapy in a large-scale randomized clinical trial. Participants in both groups achieved similar decreases in drinks per week at a 6-month follow-up, supporting the notion that digital delivery does not compromise core efficacy. Our findings extend this evidence by indicating that digital CBT can not only cut down on how much people drink but also significantly reduce how often they drink – an outcome domain sometimes presumed to benefit more from in-person contact. The fact that digital CBT in our review had a measurable impact on drinking frequency underscores its therapeutic potency across multiple usage measures.

In addition, subgroup analyses revealed that treatment goal modified the effects of face-to-face CBT on drinking quantity. Active treatment trials showed significant reductions, whereas relapse prevention trials did not, likely reflecting high baseline abstinence in those samples. This pattern suggests that the apparent lack of effect in face-to-face CBT was driven by relapse prevention contexts. Accordingly, future studies should clearly distinguish between active treatment and aftercare interventions when evaluating their efficacy.

It is important to note that some nuances in efficacy between digital and face-to-face modalities have been observed in prior research, which helps contextualize our findings. Earlier reviews have occasionally reported small advantages of face-to-face interventions for certain outcomes. For instance, a meta-analysis of brief alcohol interventions with college students found that, while both face-to-face and computer-delivered formats were effective in reducing drinking in the short term, face-to-face interventions tended to produce risk reduction across several drinking outcomes and with longer-lasting effects (Kaner et al., 2007). Direct head-to-head comparisons in some studies have also slightly favored face-to-face therapy on specific metrics (e.g. heavier drinking occasions) (Sundström et al., 2017). In our analysis, face-to-face CBT’s larger effect size on drinking frequency is consistent with these patterns, suggesting that traditional therapy may have an edge in curbing habitual or frequent drinking behaviors. However, the overall differences in efficacy were modest. A recent Cochrane review of internet-based versus in-person alcohol interventions revealed no significant difference in alcohol consumption outcomes between the two modalities (Kaner et al., 2018), in line with our observation that digital CBT can perform nearly as well as face-to-face CBT. The minor discrepancies reported across studies are likely due to the differences in populations (e.g. college students vs. clinical samples), intervention content and intensity, and the rapidly improving quality of digital interventions over the past decade. In light of more modern, well-designed digital programs, which often incorporate interactive elements and some level of human support, the gap in effectiveness between digital and face-to-face CBT appears to have narrowed substantially.

The higher proportion of women in the digital CBT group is noteworthy. This trend aligns with the rising prevalence of alcohol use among women (WHO, 2024) and reflects the well-documented barriers that women face in accessing traditional face-to-face treatment, such as caregiving responsibilities, scheduling constraints, and stigma (Levine et al., 2023). Digital delivery formats may help to mitigate these barriers, thereby supporting broader engagement and treatment uptake among women.

These findings have significant implications for treatment provision in AUD. Demonstrating that digital CBT can effectively reduce alcohol consumption and frequency means that evidence-based care can be extended beyond traditional clinic settings. A key advantage of digital CBT is the vastly improved accessibility. Individuals who are unable or reluctant to attend face-to-face therapy can engage with digital programs from home and on their own schedule. Recent epidemiological data highlight that global prevalence and mortality related to alcohol-related liver disease and AUD remain high and undertreated, particularly in low-resource settings (Danpanichkul et al., 2024; WHO, 2024). Internet and mobile-based CBT platforms can help overcome stigma and geographic barriers by providing private, on-demand support that does not require visiting a specialized clinic (White et al., 2010). Consequently, people who might never seek in-person help, due to embarrassment or a lack of nearby resources, could be reached through digital interventions. This expanded reach has the potential to facilitate early intervention in the course of problematic drinking and reduce alcohol-related harm at the population level.

Besides improving access, digital CBT offers benefits in terms of resource efficiency and scalability for healthcare systems. Once developed, digital interventions (e.g. guided self-help programs or apps) can be disseminated to large numbers of patients at a relatively low incremental cost (Riper, Blankers, et al., 2014b). Therapists can manage a larger caseload by supporting patients online (e.g. via messaging or occasional calls), or fully automated programs can deliver standardized CBT without direct therapist involvement. This efficiency is valuable for health systems constrained by workforce limitations (e.g. rural areas or low-resource settings where trained addiction counselors are scarce). Incorporating digital CBT as a complement or alternative to face-to-face therapy could allow clinics to stratify care: patients with milder alcohol misuse might start with a digital program, which allows intensive in-person resources to be reserved for more complex cases. Such stepped- or blended-care models leverage the strengths of both modalities (Sundström et al., 2017; White et al., 2010). Digital CBT could serve as a first-line intervention that provides immediate help and funnels individuals into face-to-face treatment if needed (e.g. if they do not improve with the digital option alone). Conversely, face-to-face therapy can be augmented with digital tools between sessions to reinforce skills. Ultimately, the validation of digital CBT’s efficacy means clinicians and policymakers can be more confident in deploying these tools widely, potentially increasing treatment uptake and alleviating the burden on traditional services without sacrificing the quality of care.

Despite the encouraging results, this review has several limitations that must be acknowledged. First, the number of studies directly comparing digital and face-to-face CBT was limited, and the evidence base showed substantial heterogeneity. The digital interventions evaluated varied considerably in format (e.g. web-based modules, smartphone apps, and therapist-guided vs. fully automated programs) and intensity, while the face-to-face CBT interventions also differed in session number, duration, and whether they included adjunct techniques (e.g. motivational enhancement). This variability introduces uncertainty when generalizing our findings – all digital CBT programs and traditional CBT implementations are not identical (Sundström et al., 2017). Second, outcomes were often measured via self-report (e.g. self-reported drinks per week or days of use), which can be prone to bias such as underreporting due to social desirability or recall errors (Tait & Christensen, 2010). Although such measures are standard in alcohol research, future studies should, where possible, include objective or corroborative measures (e.g. biomarker indicators of alcohol use) to strengthen confidence in the results. Third, the follow-up duration posed a challenge in many studies. Most trials assessed outcomes immediately post-treatment or at short-term follow-ups (e.g. 3 or 6 months). Thus, whether the observed reductions – especially those achieved via digital CBT – are maintained in the long term (12 months or beyond) remains unclear. Recent 2-year follow-up findings by Sundström et al. (2023) suggest that higher-intensity digital CBT programs yield more durable effects, underscoring the need to calibrate digital interventions beyond minimal contact models. Some prior data suggest that the effects of brief interventions can diminish over time (Kaner et al., 2007), so ongoing engagement or booster sessions (digital or in-person) might be necessary to sustain gains. Additionally, there may be a selection bias in who chooses digital versus in-person therapy. Participants who enroll in digital programs might differ in motivation or severity from those in face-to-face therapy (e.g. those with very severe AUD or comorbid mental health conditions might be underrepresented in digital trials) (Tait & Christensen, 2010). This could influence outcome patterns, limiting the direct comparability of groups. Finally, while our review focused on alcohol consumption metrics, we did not comprehensively examine other important outcomes such as psychosocial functioning, quality of life, or adverse events; differences in these dimensions between digital and face-to-face CBT must be explored.

These limitations highlight clear directions for future research. To build on current evidence, more head-to-head trials with robust designs are needed, particularly studies that randomly assign participants to digital versus face-to-face CBT and follow them for extended periods. Such trials would help confirm the comparative efficacy we observed and detect any emerging differences in longer-term relapse rates or health outcomes. Future studies should also seek to identify moderators of treatment response; for example, are there particular subgroups of patients for whom digital CBT works especially well (or poorly)? Characteristics such as age, baseline severity of drinking, technical literacy, or presence of social support may influence whether a person benefits more from an online format or from in-person therapy. Understanding these factors could facilitate a personalized approach to choosing the modality of intervention. Additionally, research on blended interventions – where digital and face-to-face components are combined – could capitalize on the strengths of each intervention (Sundström et al., 2017; Tait et al., 2013). Preliminary evidence suggests that blended care can be acceptable, but its impact on outcomes and cost-effectiveness warrants further study. Moreover, strategies to enhance engagement with digital CBT must be investigated, as attrition can be higher without the obligation of appointments. This might include incorporating game-like elements, periodic clinician check-ins, or peer support features in digital platforms to maintain user adherence. Finally, economic evaluations should accompany efficacy trials to quantify the cost savings (if any) of digital delivery relative to traditional therapy because this is crucial for policymakers considering large-scale implementation.

## Conclusions

In this systematic review, we compared the effects of digital and conventional face-to-face CBT on alcohol consumption outcomes. The findings indicated that digital CBT significantly reduced total alcohol intake, while both modalities effectively decreased drinking frequency, with a somewhat larger effect observed for face-to-face CBT (Andersson et al., 2014; Riper, Andersson, et al., 2014a). These results supported the hypothesis that digital CBT does not substantially underperform relative to traditional formats.

Recent large-scale trials have confirmed the noninferiority of digital CBT compared with in-person therapy in reducing alcohol use (Kiluk et al., 2024), and longer-term follow-ups suggest sustained effects with higher-intensity digital interventions (Sundström et al., 2023). Additionally, in the context of a global treatment gap – where fewer than 10% of individuals with AUD receive care – digital CBT offers a scalable, accessible alternative with public health relevance (Danpanichkul et al., 2024; WHO, 2024).

Our findings extend this evidence on interventions, holding promise for expanding treatment reach and improving outcomes across diverse populations. Importantly, our findings also indicate that treatment goal (active treatment vs. relapse prevention) may act as an effect modifier, highlighting the need to account for the intervention context when interpreting comparative effectiveness. Moreover, the higher representation of women in digital CBT studies, although not statistically significant, suggests that digital delivery formats may help overcome gender-specific access barriers, such as stigma and caregiving responsibilities, thereby supporting greater treatment uptake among women.

## Supporting information

Kim et al. supplementary material Kim et al. supplementary material

## Supplementary material

The supplementary material for this article can be found at http://doi.org/10.1017/S0033291725102043.

## References

[r1] Andersson, G., Cuijpers, P., Carlbring, P., Riper, H., & Hedman, E. (2014). Guided internet-based vs. face-to-face cognitive behavior therapy for psychiatric and somatic disorders: A systematic review and meta-analysis. World Psychiatry, 13(3), 288–295. 10.1002/wps.20151. 25273302 PMC4219070

[r2] Appleton, R., Williams, J., Vera San Juan, N., Needle, J. J., & Schlief, M. (2021). Implementation, adoption, and perceptions of telemental health during the COVID-19 pandemic: Systematic review. Journal of Medical Internet Research, 23(12), e31746. 10.2196/31746. 34709179 PMC8664153

[r3] Danpanichkul, P., Díaz, L. A., Suparan, K., Tothanarungroj, P., Sirimangklanurak, S., Auttapracha, T., … Arab, J. P. (2024). Global epidemiology of alcohol-related liver disease, liver cancer, and alcohol use disorder, 2000–2021. *Clinical and Molecular Hepatology.* 10.3350/cmh.2024.0835 PMC12016607 39957370

[r4] Higgins, J. P. T., Thomas, J., Chandler, J., Cumpston, M., Li, T., Page, M. J., & Welch, V. A. (2022). The Cochrane handbook for systematic reviews of interventions (version 6.3). Chichester, UK: Cochrane.

[r6] Kaner, E., Beyer, F., Muirhead, C., Campbell, F., Pienaar, E., Bertholet, N., … Burnand, B. (2018). Effectiveness of brief alcohol interventions in primary care populations. Cochrane Database of Systematic Reviews, 2, CD004148. 10.1002/14651858.CD004148.pub4. 29476653 PMC6491186

[r7] Kaner, E. F., Dickinson, H. O., Beyer, F. R., Campbell, F., Schlesinger, C., Heather, N., … Pienaar, E. D. (2007). Effectiveness of brief alcohol interventions in primary care populations. Cochrane Database of Systematic Reviews, 2, CD004148. 10.1002/14651858.CD004148.pub3. 17443541

[r8] Kiluk, B. D., Nich, C., Sugarman, D. E., Meshesha, L. Z., Gibbons, C. J., Martino, S., & Carroll, K. M. (2024). A digital cognitive behavioral therapy program for adults with alcohol use disorder: A randomized clinical trial. JAMA Network Open, 7(9), e2435205. 10.1001/jamanetworkopen.2024.35205. 39325452 PMC11428014

[r9] Levine, E. A., Sugarman, D. E., Rockas, M., McHugh, R. K., Jordan, C., & Greenfield, S. F. (2023). Alcohol treatment access and engagement among women in the USA: A targeted review of the literature 2012–2022. Current Addiction Reports, 10(4), 638–648. 10.1007/s40429-023-00515-1. 38505370 PMC10948108

[r10] National Institute on Alcohol Abuse and Alcoholism. (2021). Treatment for alcohol problems: Finding and getting help (NIH Publication No. 21-AA-8011). U.S. Department of Health and Human Services. https://www.niaaa.nih.gov/publications/treatment-alcohol-problems-finding-and-getting-help

[r11] Page, M. J., McKenzie, J. E., Bossuyt, P. M., Boutron, I., Hoffmann, T. C., Mulrow, C. D., … Moher, D. (2021). The PRISMA 2020 statement: An updated guideline for reporting systematic reviews. BMJ, 372(n71). 10.1136/bmj.n71. PMC8005924 33782057

[r12] R Foundation for Statistical Computing. (2023). R: A language and environment for statistical computing. Vienna, Austria: R Foundation for Statistical Computing. https://www.R-project.org

[r13] Riper, H., Andersson, G., Hunter, S. B., de Wit, J., Berking, M., Cuijpers, P., & Klein, A. (2014a). Treatment of comorbid alcohol use disorders and depression with cognitive-behavioural therapy and motivational interviewing: A meta-analysis. Addiction, 109(3), 394–406. 10.1111/add.12441. 24304463 PMC4227588

[r14] Riper, H., Blankers, M., Hadiwijaya, H., Cunningham, J., Clarke, S., Wiers, R., … Cuijpers, P. (2014b). Effectiveness of guided and unguided low-intensity internet interventions for adult alcohol misuse: A meta-analysis. PLoS One, 9(6), e99912. 10.1371/journal.pone.0099912. 24937483 PMC4061051

[r15] Riper, H., Spek, V., Boon, B., Conijn, B., Kramer, J., Martin-Abello, K., & Smit, F. (2011). Effectiveness of E-self-help interventions for curbing adult problem drinking: A meta-analysis. Journal of Medical Internet Research, 13(2), e42. 10.2196/jmir.1691. 21719411 PMC3221381

[r16] Sterne, J. A. C., Savović, J., Page, M. J., Elbers, R. G., Blencowe, N. S., Boutron, I., … Harvey, R. J. (2019). RoB 2: A revised Cochrane risk-of-bias tool for randomized trials. BMJ, 366, l4898. 10.1136/bmj.l4898. 31462531

[r17] Sundström, C., Blankers, M., & Khadjesari, Z. (2017). Computer-based interventions for problematic alcohol use: A review of systematic reviews. International Journal of Behavioral Medicine, 24(5), 646–658. 10.1007/s12529-017-9641-8. 27757844 PMC5608865

[r18] Sundström, C., Eék, N., Kraepelien, M., Lundgren, J., Kaldo, V., & Berman, A. H. (2023). High- versus low-intensity internet interventions for alcohol use disorders (AUD): A two-year follow-up of a single-blind randomized controlled trial. Internet Interventions, 33, 100630. 10.1016/j.invent.2023.100630. 37293578 PMC10244691

[r19] Sundström, C., Gajecki, M., Johansson, M., & Berman, A. H. (2021). Effects of internet-based cognitive behavioral therapy for harmful alcohol use and alcohol dependence: A randomized controlled trial. Journal of Medical Internet Research, 23(11), e29666. 10.2196/29666. 34821563 PMC8663526

[r20] Sundström, C., Peynenburg, V., Chadwick, C., Thiessen, D., Wilhelms, A., Nugent, M., … Hadjistavropoulos, H. D. (2022). Optimizing internet-delivered cognitive behaviour therapy for alcohol misuse – A randomized factorial trial examining effects of a pre-treatment assessment interview and guidance. Addiction Science & Clinical Practice, 17(1), 37. 10.1186/s13722-022-00319-0. 35871010 PMC9308037

[r21] Tait, R. J., & Christensen, H. (2010). Internet-based interventions for young people with problematic substance use: A systematic review. Medical Journal of Australia, 192(11 Suppl), S15–S21. 10.5694/j.1326-5377.2010.tb03686.x. 20528701

[r22] Tait, R. J., Spijkerman, R., & Riper, H. (2013). Internet and computer-based interventions for cannabis use: A meta-analysis. Drug and Alcohol Dependence, 133(2), 295–304. 10.1016/j.drugalcdep.2013.05.012. 23747236

[r23] White, A., Kavanagh, D., Stallman, H., Klein, B., Kay-Lambkin, F., Proudfoot, J., … Young, R. (2010). Online alcohol interventions: A systematic review. Journal of Medical Internet Research, 12(5), e62. 10.2196/jmir.1460. 21169175 PMC3057310

[r24] World Health Organization. (2024). Global status report on alcohol and health and treatment of substance use disorders. Geneva, Switzerland: World Health Organization. https://www.who.int/publications/i/item/9789240096745

