# Comparative Effectiveness of Digital Versus Face-to-Face Cognitive Behavioral Therapy for Alcohol Use Disorder: A Systematic Review and Meta-Analysis – CORRIGENDUM

**DOI:** 10.1017/S0033291725102870

**Published:** 2026-02-11

**Authors:** Ji Eun Kim, Jiyeong Kim, Nayeon Choi, Sang Kyu Lee, Hong Seok Oh, Sungwon Roh

When this article was originally published in Psychological Medicine, it omitted to include some information regarding its funding. This has been updated in the original article.

The authors apologise for this error.

## References

[r1] Kim, J. E., Kim, J., Choi, N., Lee, S. K., Oh, H. S., & Roh, S. (2025). Comparative effectiveness of digital versus face-to-face cognitive behavioral therapy for alcohol use disorder: a systematic review and meta-analysis. Psychological Medicine, 55, e315. 10.1017/S0033291725102043. 41114436 PMC12551579

