# Supplementary material for: Comparative effectiveness of digital versus face-to-face cognitive behavioral therapy for alcohol use disorder: a systematic review and meta-analysis
Source: Psychol Med. 2025 Oct 20;55:e315. doi: 10.1017/S0033291725102043 (PMC12551579; doi:10.1017/S0033291725102043)
Supplement: Kim et al. supplementary material [file S0033291725102043sup001.zip › S0033291725102043sup007.docx]

Supplementary Table S2. Study-level characteristics of included trials

| No. | First author,  Year | Type | Country | Sample size (*N*) | Recruitment period (months) | Number of sessions | Treatment goal type**** |
| --- | --- | --- | --- | --- | --- | --- | --- |
| 1 | Deady, 2016 | Digital | Australia | 60 | 6 | 4 | Active |
| 2 | Johansson, 2021 | Digital | Sweden | 692 | 25 | 12 | Active |
| 3 | Wilks, 2018 | Digital | USA | 30 | - | 8 | Active |
| 4 | Laurens, 2023 | Digital | The Netherlands | 72 | - | 5 | Active |
| 5 | Blankers, 2011 | Digital | The Netherlands | 68 | 13 | 7 | Active |
| 6 | Possemato, 2019 | Digital | USA | 15 | - | 12 | Active |
| 7 | Kacmarek, 2021 | Digital | USA | 22 | 34 | 8 | Active |
| 8 | Hyland, 2023 | Digital | Sweden | 132 | 26 | 8 | Active |
| 9 | Cougle, 2017 | Digital | USA | 30 | - | 5-8* | Active |
| 10 | Glasner, 2020 | Digital | USA | 17 | 24 | 1** | Active |
| 11 | Eek, 2023 | Digital | Sweden | 72 | 27.73 | 13 | Active |
| 12 | Zgierska, 2019 | Face-to-face | USA | 57 | - | 8 | Active |
| 13 | Baker, 2014 | Face-to-face | Australia | 60 | - | 10 | Active |
| 14 | Shakeshaft, 2002 | Face-to-face | Australia | 54 | - | 6 | Active |
| 15 | Sannibale, 2013 | Face-to-face | Australia | 29 | 25 | 12 | Active |
| 16 | Marques, 2001 | Face-to-face | Brazil | 77 | 25 | 17 | Active |
| 17 | Vedel, 2008 | Face-to-face | The Netherlands | 34 | - | 10 | Active |
| 18 | Morgenstern, 2007 | Face-to-face | USA | 47 | - | 12 | Active |
| 19 | Kushner, 2013 | Face-to-face | USA | 171 | 48 | 6 | Aftercare |
| 20 | Sitharthan, 1997 | Face-to-face | Australia | 20 | - | 6 | Active |
| 21 | Kelly, 2000 | Face-to-face | Australia | 16 | - | 6 | Active |
| 22 | Morley, 2016 | Face-to-face | Australia | 21 | - | 7-10*** | Aftercare |
| 23 | Kivlahan, 1990 | Face-to-face | USA | 15 | - | 8 | Active |
| 24 | Davis, 2016 | Face-to-face | USA | 225 | - | 12 | Active |
| 25 | Coriale, 2019 | Face-to-face | Italy | 43 | - | 5 | Aftercare |

*Note.*

*The iCBT program, delivered via a fully automated and therapist-free platform, consists of 5 core and 3 optional modules, each equivalent to one treatment session, with participants completing modules at their own pace for a total of 5 to 8 sessions.

** The ALC-TXT-CBT intervention included a single face-to-face CBT session delivered by a clinician, followed by 12 weeks of automated, personalized daily text messages. Although only one formal session occurred, thematic CBT-based content was delivered weekly via messages; these were not counted as discrete sessions due to their asynchronous and indirect format.

***The usual care condition consisted of brief, motivational enhancement-based counselling delivered over a 3-week alcohol stabilization period, with no fixed number of sessions reported. The integrated CBT condition included 7 to 10 structured sessions targeting alcohol use and comorbid mood or anxiety disorders. Both interventions were delivered at treatment initiation and were classified as initial treatments.

****Treatment goal type: Aftercare refers to studies in which participants were abstinent at baseline (≥1 day) or post-detoxification, not necessarily post-treatment maintenance. Active refers to studies without an explicit abstinence requirement or inpatient status at baseline.
