# Supplementary material for: Comparative effectiveness of digital versus face-to-face cognitive behavioral therapy for alcohol use disorder: a systematic review and meta-analysis
Source: Psychol Med. 2025 Oct 20;55:e315. doi: 10.1017/S0033291725102043 (PMC12551579; doi:10.1017/S0033291725102043)
Supplement: Kim et al. supplementary material [file S0033291725102043sup001.zip › S0033291725102043sup005.docx]

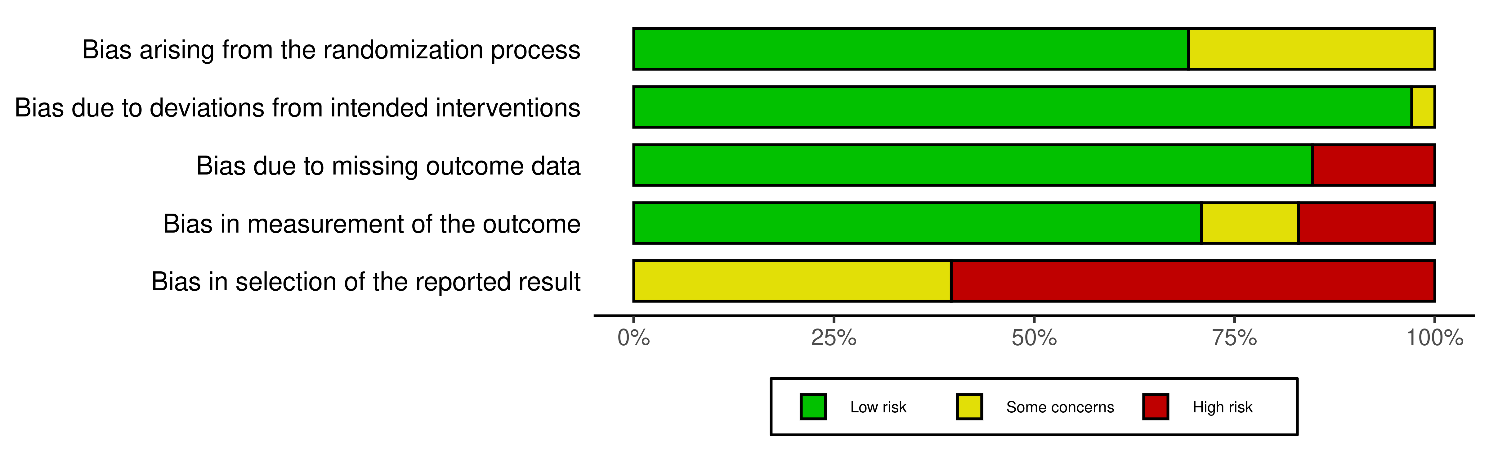
 Supplementary Figure 1. Proportion of risk of bias levels by domain


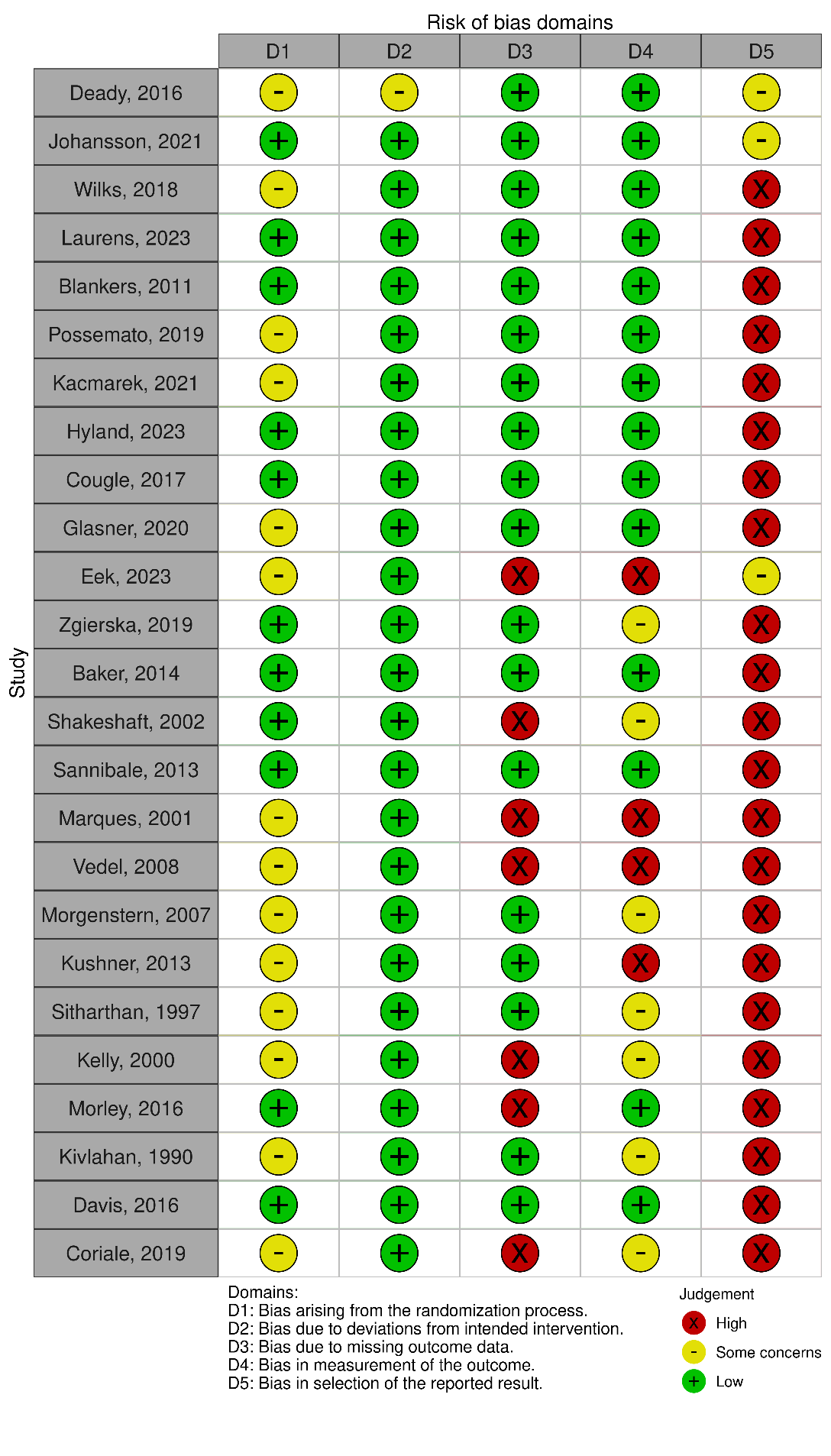


Supplementary Figure 2. Risk of bias judgments by domain for individual studies


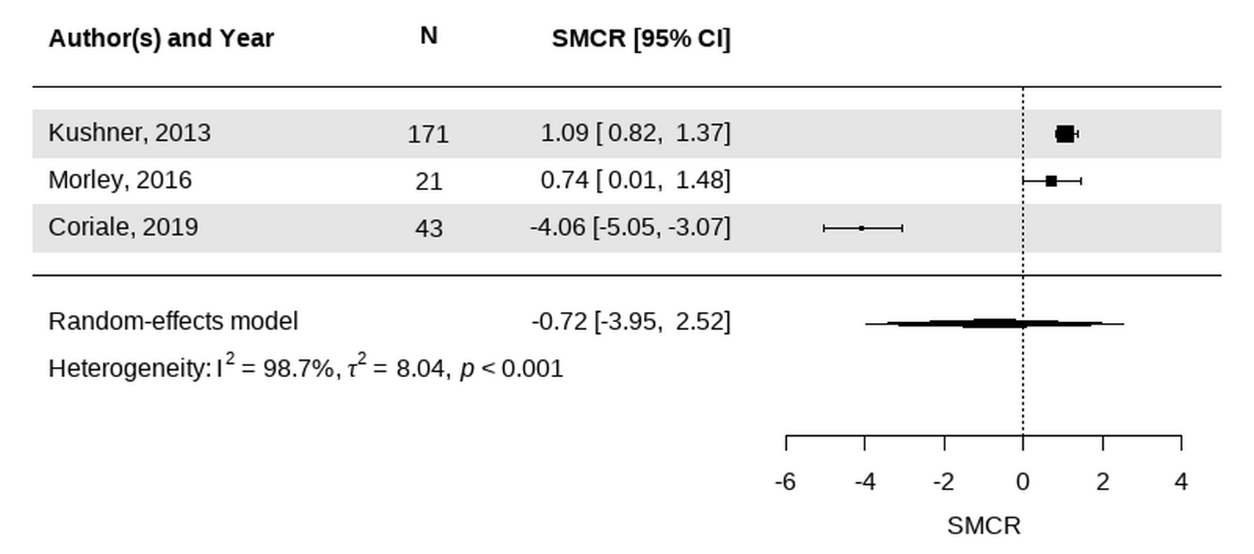


Supplementary Figure 3. Subgroup analysis: Forest plot of relapse prevention (aftercare) and CBT intervention.


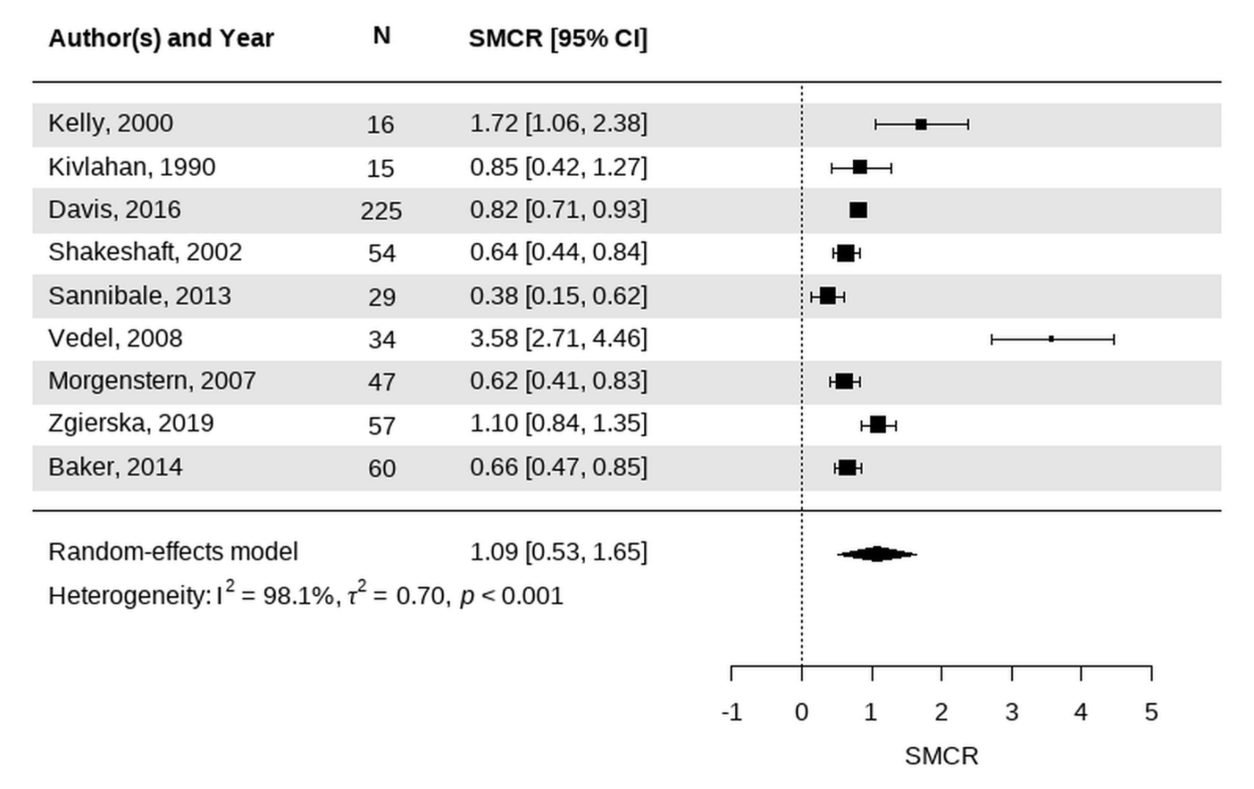


Supplementary Figure 4. Subgroup analysis: forest plot of active treatment with CBT interventions.
