# Supplementary material for: Comparative effectiveness of digital versus face-to-face cognitive behavioral therapy for alcohol use disorder: a systematic review and meta-analysis
Source: Psychol Med. 2025 Oct 20;55:e315. doi: 10.1017/S0033291725102043 (PMC12551579; doi:10.1017/S0033291725102043)
Supplement: Kim et al. supplementary material [file S0033291725102043sup001.zip › S0033291725102043sup006.docx]

Supplementary Table S1. Included studies with corresponding references

| 1 | Deady, M., Mills, K. L., Teesson, M., & Kay-Lambkin, F. (2016). An online intervention for co-occurring depression and problematic alcohol use in young people: Primary outcomes from a randomized controlled trial. *Journal of Medical Internet Research, 18*(3), e71. doi:10.2196/jmir.5178 |
| --- | --- |
| 2 | Johansson, M., Berman, A. H., Sinadinovic, K., Lindner, P., Hermansson, U., & Andréasson, S. (2021). Effects of internet-based cognitive behavioral therapy for harmful alcohol use and alcohol dependence as self-help or with therapist guidance: Three-armed randomized trial. *Journal of Medical Internet Research*, *23*(11), e29666. doi:10.2196/29666 |
| 3 | Wilks, C. R., Lungu, A., Ang, S. Y., Matsumiya, B., Yin, Q., & Linehan, M. M. (2018). A randomized controlled trial of an internet-delivered dialectical behavior therapy skills training for suicidal and heavy episodic drinkers. *Journal of Affective Disorders*, *232*, 219–228. doi:10.1016/j.jad.2018.02.053 |
| 4 | Laurens, M. C., Postel, M. G., Brusse-Keizer, M., Pieterse, M. E., Ben Allouch, S., Bohlmeijer, E. T., & Salemink, E. (2023). Augmenting outpatient alcohol treatment as usual with online approach bias modification training: A double-blind randomized controlled trial. *Addictive Behaviors*, *142*, 107630. doi:10.1016/j.addbeh.2023.107630 |
| 5 | Blankers, M., Koeter, M. W. J., & Schippers, G. M. (2011). Internet therapy versus internet self-help versus no treatment for problematic alcohol use: A randomized controlled trial. *Journal of Consulting and Clinical Psychology*, *79*(3), 330–341. doi:10.1037/a0023498 |
| 6 | Possemato, K., Johnson, E. M., Emery, J. B., Wade, M., Acosta, M. C., Marsch, L. A., Rosenblum, A., & Maisto, S. A. (2019). A pilot study comparing peer-supported web-based CBT to self-managed web CBT for primary care veterans with PTSD and hazardous alcohol use. *Psychiatric Rehabilitation Journal*, *42*(3), 305–313. doi:10.1037/prj0000334 |
| 7 | Kacmarek, C. N., Yates, B. T., Nich, C., & Kiluk, B. D. (2021). A pilot economic evaluation of computerized cognitive behavioral therapy for alcohol use disorder as an addition and alternative to traditional therapy. *Alcoholism: Clinical and Experimental Research*, *45*(5), 1109–1121. doi:10.1111/acer.14601 |
| 8 | Hyland, K., Hammarberg, A., Hedman-Lagerlöf, E., Johansson, M., Lindner, P., & Andreasson, S. (2023). The efficacy of an internet-based cognitive behavioral program added to treatment-as-usual for alcohol-dependent patients in primary care: A randomized controlled trial. *Addiction*, *118*(7), 1232–1243. doi:10.1111/add.16157 |
| 9 | Cougle, J. R., Summers, B. J., Allan, N. P., Dillon, K. H., Smith, H. L., Okey, S. A., ... & Buckner, J. D. (2017). Hostile interpretation training for individuals with alcohol use disorder and elevated trait anger: A controlled trial of a web-based intervention. *Behaviour Research and Therapy, 99*, 57–66. doi:10.1016/j.brat.2017.09.004 |
| 10 | Glasner, S., Chokron Garneau, H., Ang, A., Ray, L., Venegas, A., Rawson, R., & Kalichman, S. (2020). Preliminary efficacy of a cognitive behavioral therapy text messaging intervention targeting alcohol use and antiretroviral therapy adherence: A randomized clinical trial. *PLOS ONE*, *15*(3), e0229557. doi:10.1371/journal.pone.0229557 |
| 11 | Eék, N., Sundström, C., Kraepelien, M., Lundgren, J., Kaldo, V., & Berman, A. H. (2023). High- versus low-intensity internet interventions for alcohol use disorders (AUD): A two-year follow-up of a single-blind randomized controlled trial. *Internet Interventions*, *33*, 100630. doi:10.1016/j.invent.2023.100630 |
| 12 | Zgierska, A. E., Burzinski, C. A., Mundt, M. P., McClintock, A. S., Cox, J., Coe, C. L., Miller, M. M., & Fleming, M. F. (2019). Mindfulness-based relapse prevention for alcohol dependence: Findings from a randomized controlled trial. *Journal of Substance Abuse Treatment*, *100*, 8–17. doi:10.1016/j.jsat.2019.01.013 |
| 13 | Baker, A. L., Kavanagh, D. J., Kay-Lambkin, F. J., Hunt, S. A., Lewin, T. J., Carr, V. J., & McElduff, P. (2014). Randomized controlled trial of MICBT for co-existing alcohol misuse and depression: Outcomes to 36-months. *Journal of Substance Abuse Treatment*, *46*(3), 281–290. doi:10.1016/j.jsat.2013.10.001 |
| 14 | Shakeshaft, A. P., Bowman, J. A., & Sanson‑Fisher, R. W. (2002). Community‑based drug and alcohol counselling: Who attends and why? *Drug and Alcohol Review, 21*(2), 153–162. doi:10.1080/09595230220139055 |
| 15 | Sannibale, C., Teesson, M., Creamer, M., Sitharthan, T., Bryant, R. A., Sutherland, K., Taylor, K., Bostock-Matusko, D., Visser, A., & Peek-O’Leary, M. (2013). Randomized controlled trial of cognitive behaviour therapy for comorbid post-traumatic stress disorder and alcohol use disorders. *Addiction*, *108*(8), 1397–1410. doi:10.1111/add.12167 |
| 16 | Marques, A. C. P. R., & Formigoni, M. L. O. S. (2001). Comparison of individual and group cognitive-behavioral therapy for alcohol and/or drug-dependent patients. *Addiction*, *96*(6), 835–846. doi:10.1046/j.1360-0443.2001.9668355.x |
| 17 | Vedel, E., Emmelkamp, P. M. G., & Schippers, G. M. (2008). Individual cognitive-behavioral therapy and behavioral couples therapy in alcohol use disorder: A comparative evaluation in community-based addiction treatment centers. *Psychotherapy and Psychosomatics*, *77*(5), 280–288. doi:10.1159/000140087 |
| 18 | Morgenstern, J., Irwin, T. W., Wainberg, M. L., Parsons, J. T., Muench, F., Bux, D. A. Jr., Kahler, C. W., Marcus, S., & Schulz-Heik, J. (2007). A randomized controlled trial of goal choice interventions for alcohol use disorders among men who have sex with men. *Journal of Consulting and Clinical Psychology,* *75*(1), 72–84. doi:10.1037/0022-006X.75.1.72 |
| 19 | Kushner, M. G., Maurer, E. W., Thuras, P., Donahue, C., Frye, B., Menary, K. R., Hobbs, J., Haeny, A. M., & Van Demark, J. (2013). Hybrid cognitive behavioral therapy versus relaxation training for co-occurring anxiety and alcohol disorder: A randomized clinical trial. *Journal of Consulting and Clinical Psychology*, *81*(3), 429–442. doi:10.1037/a0031301 |
| 20 | Sitharthan, T., Sitharthan, G., Hough, M. J., & Kavanagh, D. J. (1997). Cue exposure in moderation drinking: A comparison with cognitive-behavior therapy. *Journal of Consulting and Clinical Psychology*, *65*(5), 878–882. doi:10.1037/0022-006X.65.5.878 |
| 21 | Kelly, A. B., Halford, W. K., & Young, R. M. (2000). Maritally distressed women with alcohol problems: The impact of a short-term alcohol-focused intervention on drinking behaviour and marital satisfaction. *Addiction*, *95*(10), 1537–1549. doi:10.1046/j.1360-0443.2000.951015378.x |
| 22 | Morley, K. C., Baillie, A., Leung, S., Sannibale, C., Teesson, M., & Haber, P. S. (2016). Is Specialized Integrated Treatment for Comorbid Anxiety, Depression and Alcohol Dependence Better than Treatment as Usual in a Public Hospital Setting? *Alcohol and Alcoholism*, *51*(4), 402–409. doi:10.1093/alcalc/agv131 |
| 23 | Kivlahan, D. R., Marlatt, G. A., Fromme, K., Coppel, D. B., & Williams, E. (1990). Secondary prevention with college drinkers: Evaluation of an alcohol skills training program. *Journal of Consulting and Clinical Psychology*, *58*(6), 805–810. doi:10.1037/0022-006X.58.6.805 |
| 24 | Davis, C. M., & Clifford, P. R. (2016). Ascertaining the relationships between the trajectories of specific categories of alcohol-related negative consequences and subsequent drinking behavior. *Psychology of Addictive Behaviors*, *30*(6), 648–658. doi:10.1037/adb0000181 |
| 25 | Coriale, G., De Rosa, F., Battagliese, G., Gencarelli, S., Fiore, M., Ferraguti, G., Vitali, M., Rotondo, C., Messina, M. P., Attilia, M. L., & Ceccanti, M. (2019). Motivational enhancement therapy versus cognitive behavioral therapy in a cohort of men and women with alcohol use disorder. *Biomedical Reviews*, *30*, 125–135. doi:10.14748/bmr.v30.6393 |
