# Supplementary figures and images for: Comparative effectiveness of digital versus face-to-face cognitive behavioral therapy for alcohol use disorder: a systematic review and meta-analysis
Source: Psychol Med. 2025 Oct 20;55:e315. doi: 10.1017/S0033291725102043 (PMC12551579; doi:10.1017/S0033291725102043)

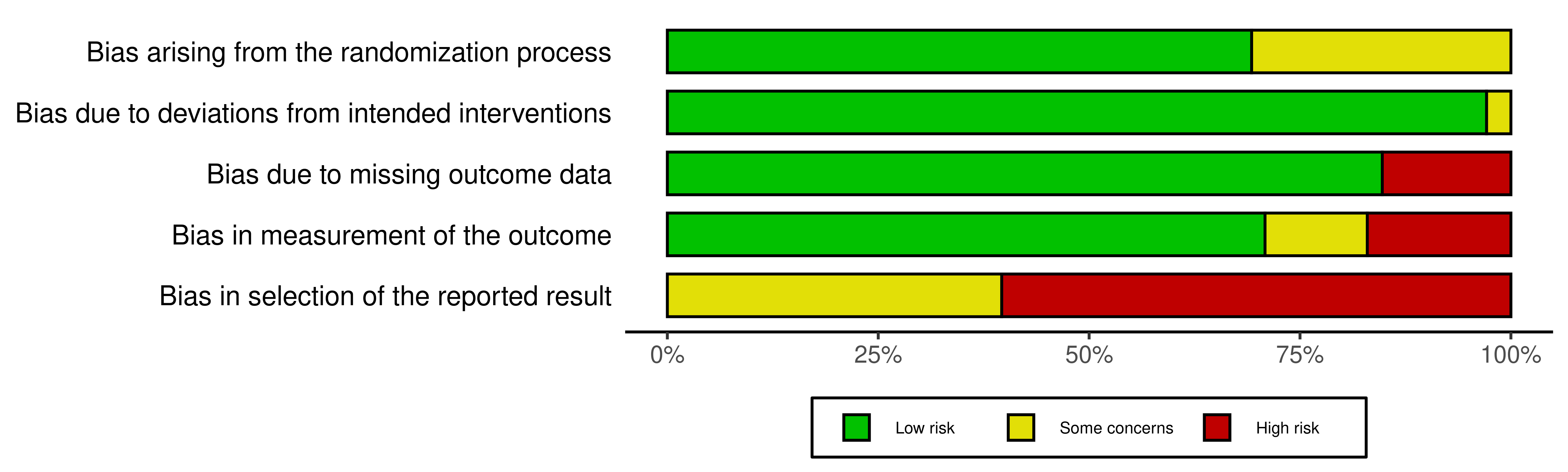

Supplement: Kim et al. supplementary material [file S0033291725102043sup001.zip › S0033291725102043sup001.tif]

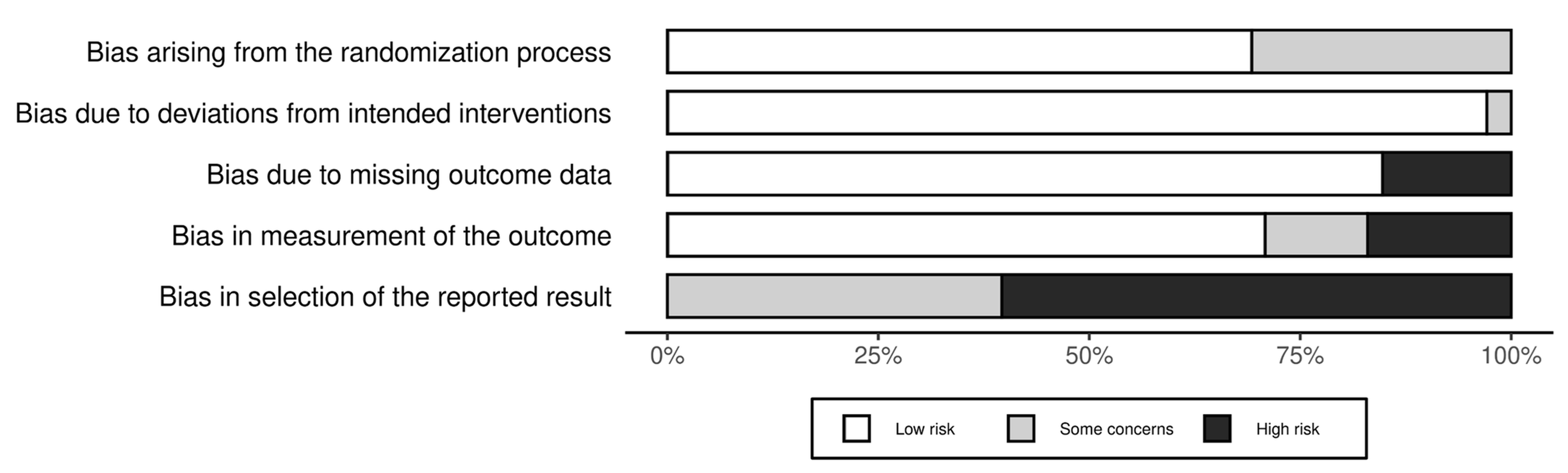

Supplement: Kim et al. supplementary material [file S0033291725102043sup001.zip › S0033291725102043sup002.tif]

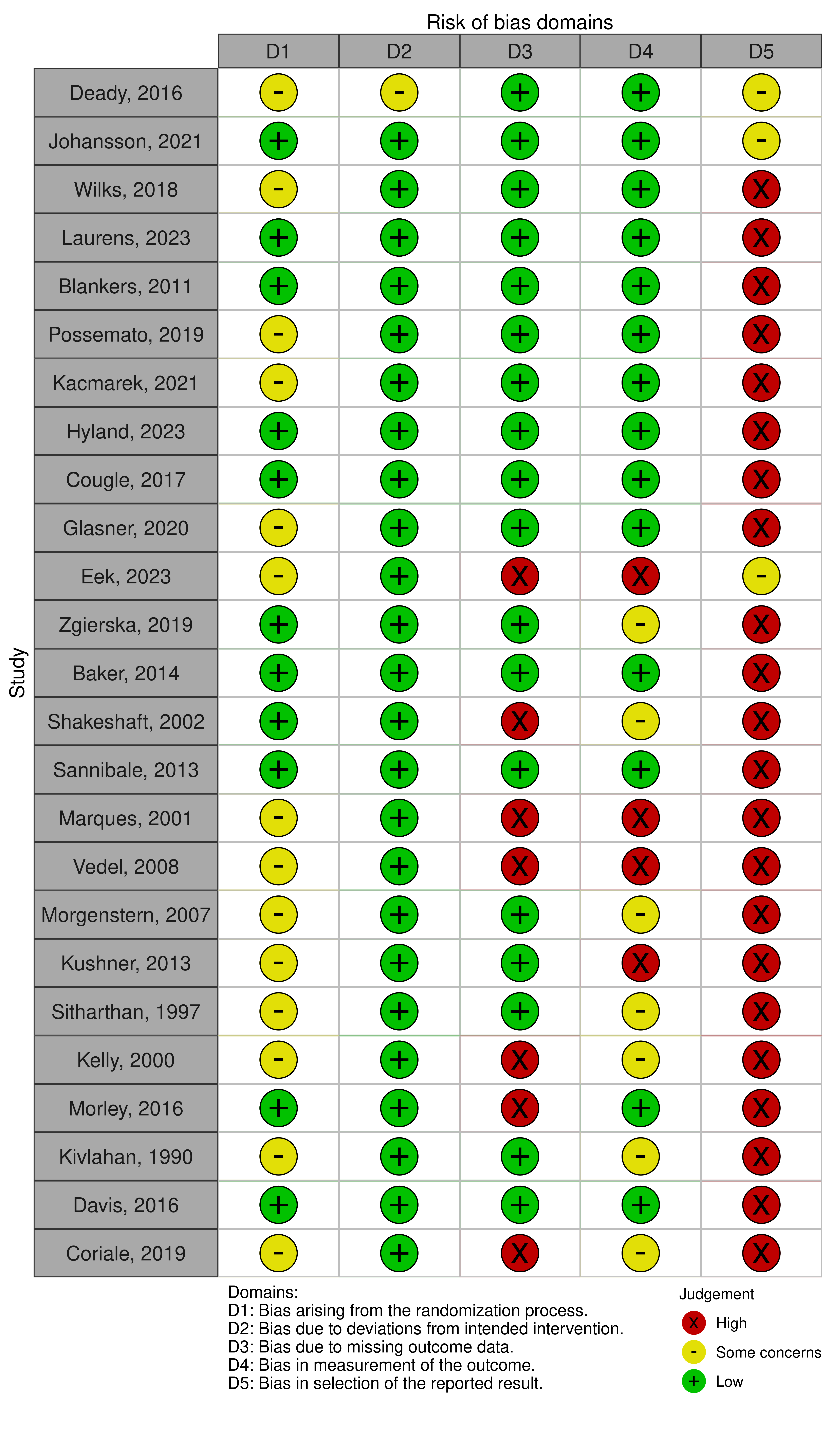

Supplement: Kim et al. supplementary material [file S0033291725102043sup001.zip › S0033291725102043sup003.tif]

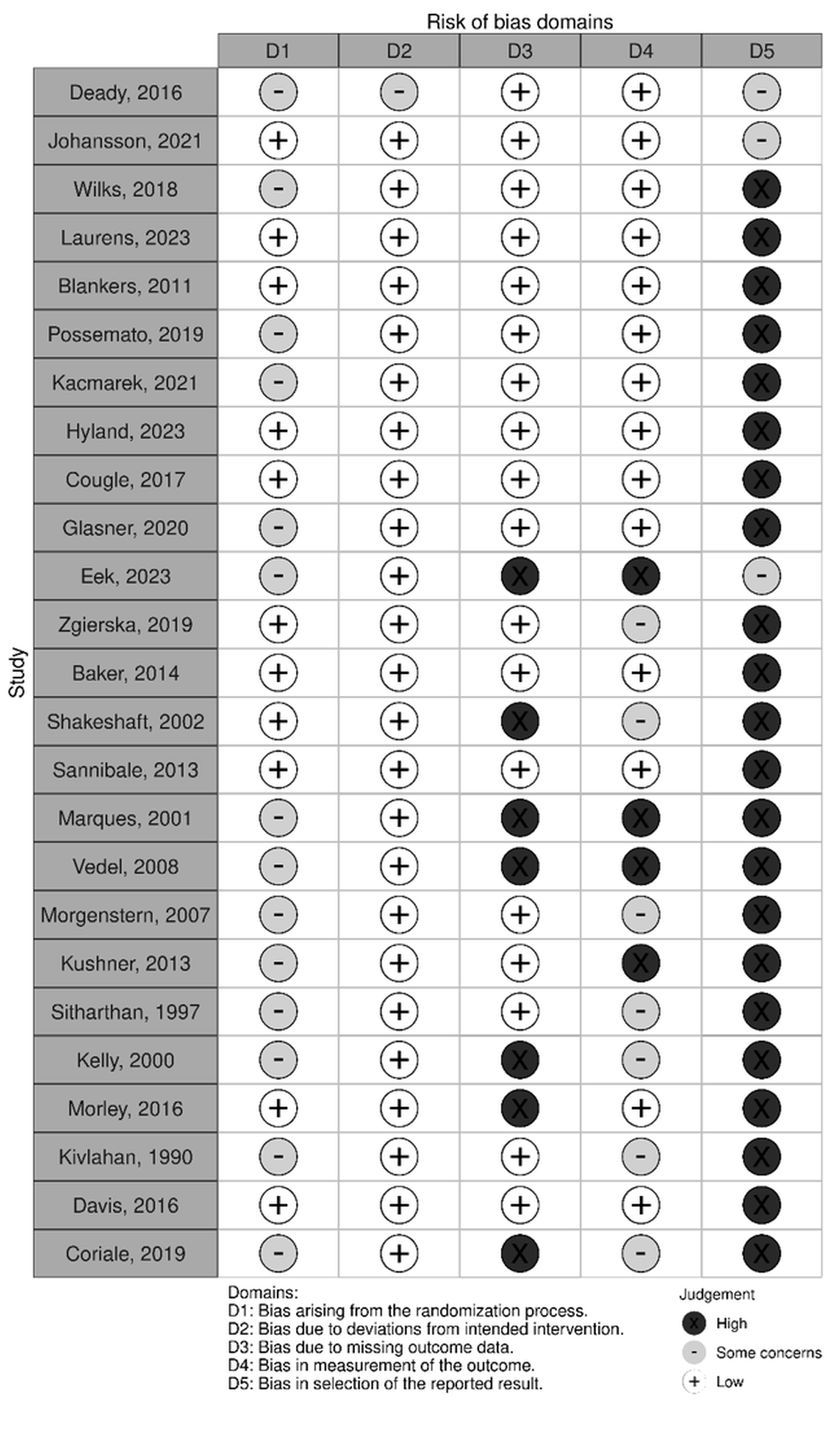

Supplement: Kim et al. supplementary material [file S0033291725102043sup001.zip › S0033291725102043sup004.tif]
